# Peroxiredoxins in disease biology: molecular mechanisms and therapeutic opportunities

**DOI:** 10.3389/fmolb.2026.1955614

**Published:** 2026-10-15

**Authors:** Yunjia Jiang, Xin Yao, Xuqi Sun, Xiaotong Wang, Ping Xia, Minggang Wei

**Affiliations:** The First Affiliated Hospital of Soochow University, Suzhou, Jiangsu, China

**Keywords:** cancer biology, ferroptosis, oxidative stress, peroxiredoxins, redox homeostasis, therapeutic targeting

## Abstract

Peroxiredoxins (PRDXs) are thiol-specific antioxidant enzymes that maintain cellular redox balance and regulate signaling pathways. They participate in the development of cancer, neurodegenerative disorders, cardiovascular diseases, inflammatory conditions, and infectious diseases. In cancer, PRDXs are frequently overexpressed and promote tumor progression, metastasis, and treatment resistance through interactions with oncogenic pathways. In neurodegenerative and cardiovascular diseases, by contrast, they support cell survival by limiting oxidative damage. Therapeutic strategies directed at PRDXs include small-molecule inhibitors, activators, and gene therapy. PRDX inhibitors sensitize tumor cells to oxidative damage, whereas PRDX activators confer neuroprotection; the principal obstacles are achieving isoform specificity and limiting off-target effects. Here we review the structure and biology of the PRDX family, its isoform- and context-dependent functions across these disease settings, and the evidence supporting PRDXs as therapeutic targets and prognostic biomarkers.

## 1 Introduction

The discovery of the peroxiredoxin (PRDX) family dates back to the early 1990s, when they were first identified as antioxidant proteins capable of reducing peroxides (Chae et al., 1994). In mammals, the PRDX family comprises six thiol-dependent peroxidases, PRDX1 to PRDX6, which swiftly reduce hydrogen peroxide, organic hydroperoxides, and peroxynitrite. Their abundance and high reactivity toward peroxides position them as major intracellular peroxide sinks, while their regulation of local H_2_O_2_ facilitates redox signaling (Winterbourn, 2025). Consequently, PRDXs should be regarded not only as antioxidant enzymes but also as compartmentalized sensors and transducers of peroxide signals (Rhee et al., 2012; Thapa et al., 2023).

This dual function renders PRDX activity context- and isoform-dependent in various diseases. The six isoforms are not functionally interchangeable; their distribution across the cytosol, nucleus, mitochondria, endoplasmic reticulum, peroxisomes, lysosomes, and extracellular space enables compartment-specific control of peroxides (Figure 1) (Kim et al., 2026). Increased expression of PRDX in numerous tumors supports survival in an oxidizing microenvironment and may promote tumor progression or treatment resistance (Wu M. et al., 2022; Thapa et al., 2022). In contrast, in neurodegenerative diseases, the loss or inactivation of specific isoforms can exacerbate oxidative injury and neuronal death (Szeliga, 2020). A similar context dependence is observed in cardiovascular, inflammatory, and infectious disorders, where PRDXs may protect host tissues while simultaneously reshaping stress and immune signaling (Li et al., 2020; Robinson et al., 2010). Therefore, the pertinent question is not whether PRDXs are broadly protective or pathogenic, but rather which isoform, cellular compartment, and catalytic state predominate in a given context.

**FIGURE 1:**
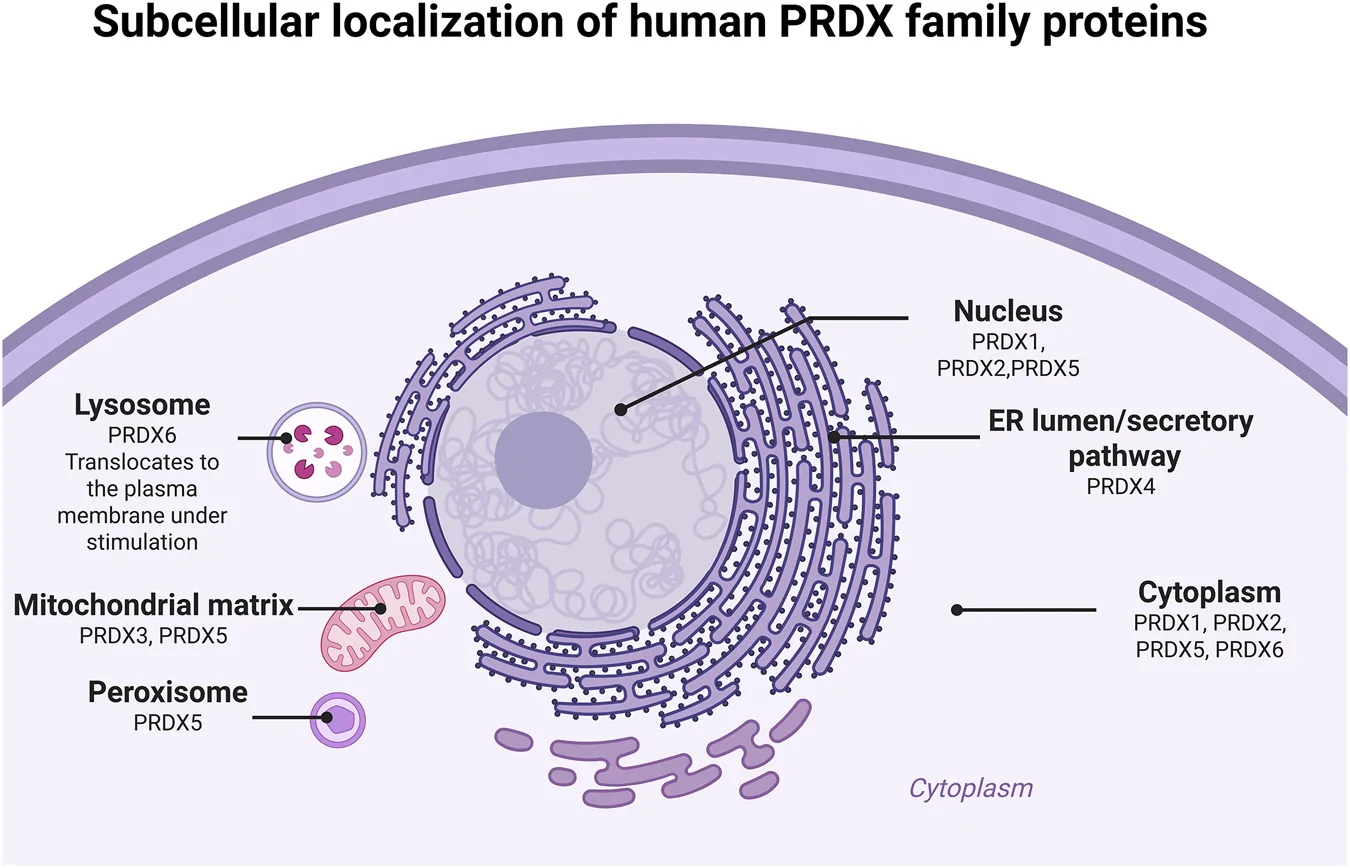
Principal subcellular localization of human PRDX family proteins. Schematic representation of the predominant subcellular distribution of the six human peroxiredoxin (PRDX) isoforms. PRDX1 and PRDX2 are localized mainly in the cytoplasm and nucleus; PRDX3 is predominantly localized to the mitochondrial matrix; PRDX4 is targeted to the endoplasmic reticulum (ER) lumen and secretory pathway; PRDX5 is distributed among the cytoplasm, nucleus, mitochondrial matrix, and peroxisomes; and PRDX6 is localized mainly in the cytoplasm and lysosomes. This compartment-specific distribution enables PRDX isoforms to coordinate peroxide detoxification and redox signaling across distinct cellular compartments.

This review begins by summarizing the genomic organization, catalytic classification, and non-peroxidase functions of the PRDX family. Subsequently, we examine the isoform-specific roles of PRDXs in various diseases, including cancer, neurodegenerative disorders, cardiovascular diseases, inflammation, and infections, while evaluating the strength of evidence for each condition. Finally, we consider the potential of PRDXs as therapeutic targets and biomarkers.

## 2 The peroxiredoxin family: organization, catalysis and functional range

### 2.1 Genomic organization and subcellular distribution

The six human PRDX genes are distributed across five chromosomes: *PRDX1* and *PRDX6* on chromosome 1, *PRDX3* on chromosome 10, *PRDX5* on chromosome 11, *PRDX2* on chromosome 19, and *PRDX4* on the X chromosome. Figure 2 summarizes their exon–intron structures and the lengths of the encoded proteins. These genes are not clustered in a manner that suggests shared transcriptional control; instead, functional divergence within the family is determined by targeting sequences and active-site architecture. The protein products occupy distinct yet partially overlapping compartments. PRDX1 and PRDX2 are primarily located in the cytosol and nucleus, while PRDX3 is concentrated in the mitochondrial matrix. PRDX4 is involved in the endoplasmic reticulum and secretory pathway, PRDX5 is distributed among the cytosol, nucleus, mitochondria, and peroxisomes, and PRDX6 is found in the cytosol and lysosomes, with the ability to translocate to membranes (Figure 1) (Kim et al., 2026; Rahaman et al., 2024). This compartmentalization has functional significance rather than being incidental: because the diffusion of H_2_O_2_ is restricted by both membranes and the peroxidases themselves, each isoform regulates peroxide levels within a distinct subcellular pool. Consequently, isoforms are poor substitutes for one another despite their catalytic similarities, a point that recurs throughout the disease literature discussed below.

**FIGURE 2:**
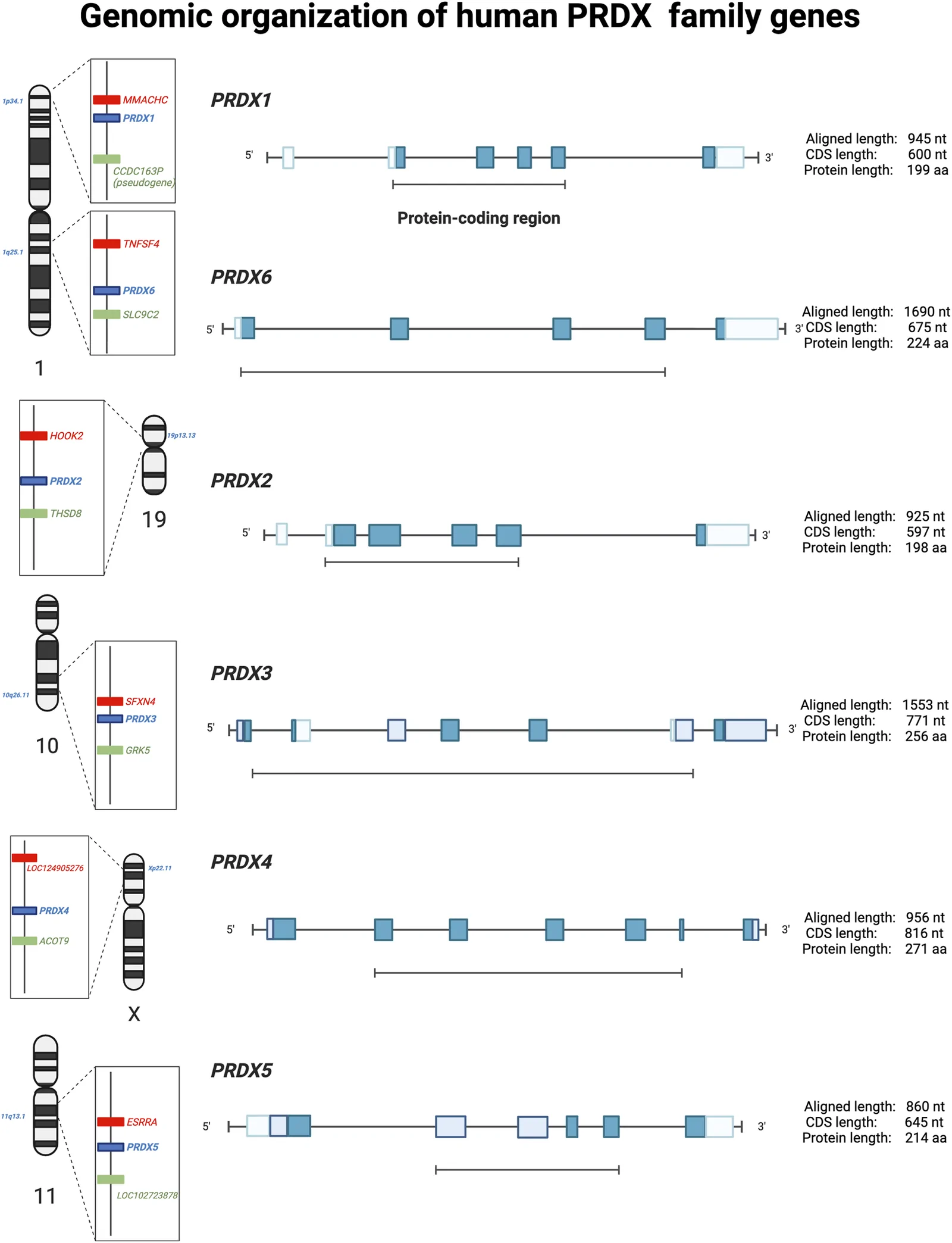
Genomic organization of human PRDX family genes. Schematic representation of the chromosomal locations and exon–intron structures of the six human PRDX genes. The chromosomal ideograms on the left indicate the cytogenetic locations and selected neighboring genes of *PRDX1* (1p34.1), *PRDX6* (1q25.1), *PRDX2* (19p13.13), *PRDX3* (10q26.11), *PRDX4* (Xp22.11), and *PRDX5* (11q13.1). The diagrams on the right show the exon–intron organization of representative transcripts, with boxes representing exons and connecting lines representing introns. The brackets beneath the transcripts indicate the protein-coding regions. Transcript length, coding sequence (CDS) length, and predicted protein length are provided for each PRDX gene. Genomic information was obtained from the NCBI Gene database. CDS, coding sequence; nt, nucleotides; aa, amino acids.

### 2.2 Catalytic classes and the significance of hyperoxidation

Despite their different locations, all PRDXs use a conserved peroxidatic cysteine to attack a peroxide substrate and form a sulfenic acid intermediate. Typical 2-Cys PRDXs (PRDX1 to PRDX4) resolve this intermediate by forming an intermolecular disulfide with the resolving cysteine of a partner subunit. Atypical 2-Cys PRDX5 instead forms an intramolecular disulfide, whereas 1-Cys PRDX6 lacks a resolving cysteine and relies on a glutathione-dependent reduction system (Nevalainen, 2010). Thioredoxin returns typical and atypical 2-Cys PRDXs to the reduced state and completes their catalytic cycle (Winterbourn, 2025; Rhee and Woo, 2020). These differences in disulfide formation and reductant use provide a mechanistic basis for the conventional classification of mammalian PRDXs into typical 2-Cys, atypical 2-Cys, and 1-Cys enzymes (Kim et al., 2026).

PRDX function is further adjusted after peroxide reduction. When peroxide flux exceeds the capacity of the catalytic cycle, the peroxidatic cysteine of 2-Cys PRDXs can be hyperoxidized to sulfinic acid and subsequently repaired by sulfiredoxin (Hayward and Baud, 2025). Hyperoxidation, other post-translational modifications, and transitions between dimers, decamers, and higher-order assemblies alter peroxidase activity, substrate access, and protein interactions (Rhee and Woo, 2020; Troussicot et al., 2021). Reversible hyperoxidation therefore represents a regulated shift in enzyme state rather than simply irreversible catalytic failure (Winterbourn, 2025).

### 2.3 Chaperone activity and redox relay

Under severe oxidative stress, selected 2-Cys PRDXs assemble into the high-molecular-weight complexes noted above, which stabilize unfolded proteins and limit their aggregation (Troussicot et al., 2021). This holdase activity is best characterized for cytosolic PRDX1 and PRDX2, whereas mitochondrial PRDX3 stacks its dodecameric toroids into filaments whose lumen carries hydrophobic patches proposed to bind unfolded clients (Radjainia et al., 2015), although a later structure argued that these assemblies mainly preserve the catalytic state of PRDX3 itself (Yewdall et al., 2016). PRDX4 also oligomerizes on oxidation and engages the endoplasmic reticulum (ER) folding machinery (Elko et al., 2021), but it reaches proteostasis by a different chemistry, delivering oxidizing equivalents to protein disulfide isomerase in a pathway parallel to endoplasmic reticulum oxidoreductin 1 (ERO1) (Fujii et al., 2025). The atypical 2-Cys PRDX5 and the 1-Cys PRDX6 do not assemble the decameric rings described above and are structurally excluded from this transition; PRDX6 moonlights instead through its calcium-independent phospholipase A_2_ activity (iPLA2), which underlies its contributions to membrane repair and lipid metabolism (Rahaman et al., 2024). At lower peroxide fluxes, PRDXs can act as redox sensors or relays, transferring oxidizing equivalents to partner proteins and thereby regulating selected signaling pathways (Rhee et al., 2012). This relay function is important because many signaling proteins react too slowly with hydrogen peroxide to account for the speed and specificity of cellular responses by direct oxidation alone (Winterbourn, 2025). The balance among peroxidase activity, chaperone assembly, and redox-relay signaling is determined by isoform, compartment, oxidation state, and interacting partners. This mechanistic understanding elucidates why the disease phenotypes reviewed below diverge markedly between isoforms.

## 3 Peroxiredoxin family in disease pathophysiology

### 3.1 PRDXs in cancer biology

PRDXs, including PRDX1 to PRDX6, have complex and sometimes paradoxical relationships with tumor initiation, progression, and metastasis (Thapa et al., 2023; Wu M. et al., 2022; Seitz et al., 2024). Their functions in cancer are diverse, with different PRDX isoforms contributing to distinct aspects of cancer cell biology, often depending on the specific tumor context and microenvironment (Thapa et al., 2023; Wu M. et al., 2022; Seitz et al., 2024). In many cancers, PRDXs are overexpressed and can support tumor growth and protect cancer cells from oxidative stress, thereby promoting survival and resistance to therapy. The following subsections distinguish functional evidence from association and summarize both the strengths and limitations of the available studies.

#### 3.1.1 PRDX1

PRDX1 is the most extensively studied family member, with evidence spanning breast, colorectal, lung, prostate and esophageal cancers (Guan et al., 2024). However, this breadth of investigation does not necessarily translate into depth of mechanistic understanding. On the contrary, the reported tumor-promoting and tumor-suppressive activities of PRDX1 in different settings make it one of the most controversial isoforms (Cao et al., 2009; Min et al., 2018).

##### 3.1.1.1 Breast cancer

The therapeutic potential of targeting PRDX1 in breast cancer was first established through functional intervention studies. Bajor et al. showed that targeting PRDX1 impairs breast cancer cell growth and sensitizes these cells to oxidative stress, supporting further evaluation of PRDX1 as a therapeutic target, especially when combined with prooxidant therapies such as pharmacological ascorbate (Bajor et al., 2018). Subsequent work strengthened this rationale from two complementary angles. Zhou et al. identified an HBXIP/NRF2 feedback loop that stabilizes PRDX1, thereby preventing JNK1-mediated apoptosis and promoting anoikis resistance, positioning PRDX1 within a regulatory network that governs redox adaptation in breast cancer (Zhou et al., 2022). Similarly, Attaran et al. identified PRDX1 as a regulator of extracellular matrix (ECM) remodeling through interactions with lysyl oxidase (LOX) (Attaran et al., 2021). The mechanistic rationale was translated into a direct pharmacological proof-of-concept by Spínola-Lasso et al. who demonstrated that the anti-tumor compound CM728 binds and inactivates PRDX1 in triple-negative breast cancer (TNBC), triggering oxygen species (ROS) accumulation, JNK/p38 MAPK activation, signal transducer and activator of transcription 3 (STAT3) inhibition, cell-cycle arrest and apoptosis (Spínola-Lasso et al., 2023). Collectively, these findings have redefined PRDX1 from a merely “targetable antioxidant enzyme” into a “core regulator linking redox adaptation, ECM remodeling and survival signaling.” Importantly, however, most of the available evidence derives from TNBC models; the role of PRDX1 in hormone-receptor-positive or HER2-positive breast cancer remains unexplored. Moreover, the current data rely predominantly on cell lines and xenografts, with limited validation in genetically engineered mouse models or patient-derived organoids. Thus, while the case for PRDX1 as a target in breast cancer has gained early support, its generalizability across breast cancer subtypes and its translational readiness remain to be established.

##### 3.1.1.2 Colorectal cancer

The clinical relevance of PRDX1 in colorectal cancer was first suggested by prognostic data. Li et al. found that high PRDX1 expression correlates with increased tumor grade, lymph node metastasis, and decreased patient survival (Li et al., 2018). PRDX1 enhances cancer cell invasion and migration by upregulating matrix metalloproteinase 2 (MMP2), matrix metalloproteinase 9 (MMP9), and vascular endothelial growth factor A (VEGFA), which are required for metastasis and angiogenesis. Building on these clinical observations, Xu et al. delineated a mechanistic pathway by which PRDX1 sustains colorectal cancer cell survival under oxidative stress: PRDX1 enhances Cullin-5 neddylation, which in turn promotes NOXA degradation and thereby suppresses apoptosis (Xu S. et al., 2021). More recently, the functional landscape of PRDX1 in colorectal cancer has expanded further. Song et al. discovered that PRDX1 acts as a molecular chaperone that stabilizes nuclear factor erythroid 2-related factor 2 (NRF2) by limiting CUL3-dependent ubiquitination, thereby sustaining glutathione peroxidase 4 (GPX4) expression and suppressing ferroptosis, an activity that extends PRDX1 function from peroxide metabolism to ferroptosis regulation (Song Y. et al., 2024). In parallel, Sun et al. found that PRDX1 exerts immunosuppressive functions in tumor-associated macrophages; targeted deletion of *Prdx1* reprograms these macrophages toward a pro-inflammatory phenotype, enhances CD4^+^ and CD8^+^ T-cell infiltration, inhibits tumor growth, and improves anti-PD-1 therapy responses (Sun et al., 2025). Thus, PRDX1 in colorectal cancer has evolved from a simple “antioxidant enzyme” into a “multi-nodal regulator linking cell survival, ferroptosis resistance and immune microenvironment remodeling.” This functional complexity makes PRDX1 an attractive therapeutic target—in principle, its inhibition might simultaneously kill tumor cells and activate anti-tumor immunity. However, the opposing functions of PRDX1 in tumor cells versus immune cells suggest that achieving therapeutic benefit may require cell-type-selective or function-selective modulation. Celastrol has been reported to covalently modify PRDX1 in colorectal cancer cells, accompanied by ROS accumulation and apoptosis (Xu et al., 2023). However, celastrol is a quinone methide triterpenoid that reacts with multiple nucleophilic cysteine residues and has documented activity against several unrelated targets; PRDX1 should therefore be regarded as one of its cellular targets rather than as a selectively inhibited one.

##### 3.1.1.3 Esophageal cancer

The evidence for PRDX1 in esophageal squamous cell carcinoma (ESCC) indicates a context-dependent role rather than a uniformly tumor-promoting or tumor-suppressive function. Loss-of-function experiments support a pro-tumorigenic activity: *PRDX1* silencing reduces ESCC cell proliferation and increases apoptosis in association with decreased PI3K/AKT activity, and miR-375-mediated repression of PRDX1 suppresses tumor growth and invasion in cellular and xenograft models (Wu K. et al., 2022; Song et al., 2019). Paradoxically, however, the histone deacetylase inhibitor romidepsin (FK228) increases PRDX1 expression through promoter-associated histone acetylation, accompanied by p21 induction and apoptosis; PRDX1 depletion attenuates this treatment response (Hoshino et al., 2005). A possible resolution to this paradox is that PRDX1 serves dual roles in ESCC—supporting basal tumor-cell fitness through its peroxidase activity under homeostatic conditions, while also participating in a drug-induced growth-suppressive program under FK228-mediated stress. This hypothesis implies that the functional direction of PRDX1 in ESCC is not fixed but depends on cellular stress state and upstream inputs. Nevertheless, these explanations have not been tested in a unified model system, and the functional duality of PRDX1 in ESCC warrants further investigation using conditional genetic approaches.

##### 3.1.1.4 Lung cancer

Studies of PRDX1 in lung cancer have focused on two broad areas: maintenance of basal malignant phenotypes and adaptation to therapeutic stress. Feng et al. showed that the super-enhancer-driven lncRNA LINC00880 acts as a scaffold bridging cyclin-dependent kinase 1 (CDK1) and PRDX1, promoting PI3K/AKT signaling and sustaining the malignant phenotype of lung adenocarcinoma (Feng et al., 2023). In the context of therapeutic stress, Hao et al. reported that under hypoxic conditions PRDX1 increases radiotherapy resistance in non-small-cell lung cancer (NSCLC) through the TLR4-NF-κB/p65 pathway (Hao J. et al., 2023), and Li et al. identified PRDX1 as a driver of resistance to ataxia-telangiectasia mutated (ATM) kinase inhibition through metabolic dependency mapping (Li H. et al., 2025). The pharmacological relevance of these observations is supported by Wang et al., who found that PRDX1-directed compounds induce ferroptotic and apoptotic death in lung cancer models (Wang Y. et al., 2025). Collectively, these findings suggest that PRDX1 helps lung cancer cells survive under conditions of hypoxia, radiotherapy and targeted therapy, making it an attractive candidate for combination therapy. However, most studies have been confined to individual cell lines and specific stress conditions. The functional heterogeneity of PRDX1 across molecular subtypes of lung cancer, particularly those defined by epidermal growth factor receptor (EGFR) and KRAS proto-oncogene, GTPase (KRAS) mutations, remains unclear, as does its potential utility for patient stratification.

##### 3.1.1.5 Prostate cancer

Research on PRDX1 in prostate cancer has progressed from tumor-cell-autonomous signaling to microenvironmental regulation and, subsequently, to molecular interaction mechanisms. At the cell-autonomous level, Park et al. found that PRDX1 directly interacts with the androgen receptor (AR) and enhances its transcriptional activity, even under hypoxia/reoxygenation conditions, suggesting that PRDX1 may contribute to sustained AR signaling in castration-resistant prostate cancer (CRPC) (Park et al., 2007). Riddell et al. extended this finding to the tumor microenvironment, showing that tumor-derived PRDX1 promotes TLR4-and VEGF-dependent endothelial responses and tumor vascularization, whereas PRDX1 inhibition reduces vascular development and tumor growth in experimental models (Riddell et al., 2011). Mechanistically, Dasari et al. further identified tumor protein D52 (TPD52) as an interaction partner that enhances PRDX1 peroxidase activity and supports prostate cancer cell growth, survival and migration (Dasari et al., 2019). Thus, PRDX1 in prostate cancer not only sustains tumor cell survival through AR signaling but also remodels the microenvironment through the TLR4/VEGF axis, and its activity is finely tuned by interacting partners such as TPD52. This multi-layered functional network nominates PRDX1 as a candidate target for combination therapy in CRPC. However, all of the available evidence is preclinical; clinical associations between PRDX1 expression or activity and patient prognosis or response to AR-targeted therapies have not been established.

##### 3.1.1.6 Cross-cancer implications

Beyond these site-specific mechanisms, metabolic dependency mapping identified PRDX1 as a determinant of resistance to ATM inhibition. *PRDX1* loss sensitized tumor cells to ATM inhibition by promoting disulfide-stress-dependent RPL32 oxidation and p53 activation (Li H. et al., 2025). Although pan-cancer dependency analyses suggested that this interaction may extend beyond one tumor type, its functional validation was centered mainly on NSCLC models and depended on functional p53. The relationship therefore cannot yet be generalized across tumor types.

Overall, the PRDX1 literature does not support a uniform directional effect in cancer; rather, the relationship depends on tumor context and disease stage. Tumor-suppressive evidence derives primarily from genetic loss models linking *Prdx1* deficiency to PTEN oxidation and AKT activation (Cao et al., 2009), whereas tumor-promoting effects emerge from functional studies in established tumors that implicate PRDX1 in redox adaptation, ferroptosis resistance, treatment resistance and stromal or vascular signaling. Human data are largely associative, and PRDX1 abundance alone cannot distinguish a driver from an adaptive marker. The apparent contradictions may reflect differences between tumor initiation and maintenance, and between catalytic and non-peroxidase functions, but these explanations have not been tested within a common model system. Future studies should systematically compare PRDX1 function across tumor stages using genetically engineered mouse models, and develop mutants that separate catalytic from non-catalytic activities to dissect the molecular basis of its functional duality.

#### 3.1.2 PRDX2

PRDX2 has been studied predominantly in colorectal cancer, gastric cancer, TNBC and cervical cancer, but its tumor-suppressive role in melanoma complicates its functional interpretation. In colorectal cancer, PRDX2 promotes cell proliferation by facilitating p53 degradation through the MDM2-RPL4-p53 pathway; PRDX2 silencing stabilizes p53 and inhibits tumor growth, suggesting its potential as a therapeutic target (Wang et al., 2021). Further, Lv et al. identified a regulatory loop involving c-Myc/miR-200b-3p/PRDX2 that promotes colorectal cancer progression and chemoresistance (Lv et al., 2017). In gastric cancer, celastrol has likewise been shown to engage PRDX2 covalently, with ROS-induced ER stress, mitochondrial dysfunction, and reduced tumor growth (Chen et al., 2020). However, celastrol is a multi-target compound, and its anti-tumor effects likely involve multiple covalent targets; it cannot be considered a selective PRDX2 inhibitor. Recent studies have reported additional tumor-associated functions of PRDX2. In TNBC, PRDX2 was significantly upregulated and promoted tumor migration, invasion, and epithelial–mesenchymal transition (EMT) through activation of the PI3K/AKT/SP1/FN1 regulatory axis. Genetic depletion or pharmacological inhibition of PRDX2 suppressed tumor growth and metastatic phenotypes, identifying PRDX2 as a potential therapeutic target in TNBC (Zhou et al., 2025). PRDX2, like PRDX1, buffers bleomycin-induced oxidative stress and attenuates ER stress and apoptosis, suggesting that PRDX2 inhibition may enhance chemosensitivity by amplifying ROS-dependent ER stress signaling (Sun et al., 2024). However, PRDX2 is not universally pro-tumorigenic. In melanoma, Lee et al. reported a striking counterexample: PRDX2 suppresses metastasis by inhibiting Src/ERK activation and stabilizing E-cadherin/β-catenin complexes at adherens junctions; its silencing promotes proliferation and migration (Lee et al., 2013). This counterexample underscores a key conceptual point: the tumor-associated functions of PRDX2 cannot be predicted from its antioxidant capacity alone, but must be understood in the context of cell-type-specific interacting partners. Thus, PRDX2 is predominantly pro-tumorigenic in colorectal cancer, gastric cancer and TNBC, but tumor-suppressive in melanoma. This contrast implies that targeting PRDX2 must be evaluated in a tumor-context-specific manner rather than as a pan-cancer strategy. Future work should focus on identifying the key interaction partners and tissue-specific modifications that determine the functional direction of PRDX2.

#### 3.1.3 PRDX3

PRDX3, primarily localized in the mitochondrial matrix, plays an essential role in regulating oxidative stress, which often contributes to tumor progression and metastasis, particularly in aggressive cancers.

##### 3.1.3.1 Breast cancer

PRDX3 in TNBC is primarily associated with promoting metastasis. Chua et al. showed that PRDX3 promotes metastasis in TNBC by upregulating MMP-1 through the ERK/AP-1 signaling pathway (Chua et al., 2024). *PRDX3* knockdown reduced MMP-1 levels and cell invasiveness, whereas overexpression enhanced these processes. Inhibition of ERK signaling significantly reduced PRDX3-mediated MMP-1 expression, suggesting that targeting this pathway may be a viable strategy against TNBC metastasis. In addition, Chen et al. demonstrated that silencing B7-H4 in breast cancer cells decreased PRDX3 expression, leading to increased ROS levels and reduced cell viability (Chen et al., 2022). Together, these two studies delineate a signaling axis from B7-H4 to PRDX3 to ERK/AP-1 to MMP-1, linking immune-regulatory signals to redox adaptation and metastatic execution. However, these findings are largely based on cell lines and xenografts, and the prognostic independence of PRDX3 in breast cancer patients awaits validation in large cohorts.

##### 3.1.3.2 Gastric cancer

Zhao et al. identified the FOXM1/ASF1B/PRDX3 axis as a driver of gastric cancer progression, with FOXM1 promoting PRDX3 expression via ASF1B, thereby increasing resistance to oxidative stress (Zhao et al., 2024). Perturbation of this pathway suppressed tumor growth and increased oxidative-stress sensitivity in experimental models, supporting a preclinical target hypothesis.

##### 3.1.3.3 Pancreatic cancer

Recent studies have identified PRDX3 as a potential prognostic biomarker in pancreatic ductal adenocarcinoma (PDAC). Elevated PRDX3 expression was associated with tumor progression and poorer survival, suggesting a role in oxidative stress adaptation during PDAC development (Kakehashi et al., 2025). Mechanistically, SALL4 suppresses mitochondrial ROS in PDAC through a FOXM1/PRDX3 axis, thereby promoting epithelial–mesenchymal transition and metastatic capacity (Huynh et al., 2018). FOXM1 recurs as an upstream regulator of PRDX3 in gastric cancer, pancreatic cancer, and stem-like tumor populations.

##### 3.1.3.4 Glioblastoma

Huang et al. examined the role of PRDX3 in glioblastoma stem-like cells, where it suppresses mitochondrial ROS and promotes self-renewal (Huang et al., 2021). Disrupting the prohibitin (PHB)–PRDX3 axis enhanced radiosensitivity in experimental models, nominating this pathway for further study without establishing clinical target validity.

##### 3.1.3.5 Endometrial and colorectal cancer stem cells

PRDX3 was enriched in stem-like tumor-cell populations in endometrial and colorectal cancer and was linked to FOXM1-dependent maintenance of mitochondrial function, sphere formation, survival, xenograft growth, and metastatic capacity (Song et al., 2017; Song et al., 2015). These studies extend the PRDX3 survival phenotype to cancer stem-like cells, although they remain based on selected experimental populations rather than unselected human tumors.

##### 3.1.3.6 Pleural mesothelioma

Genetic deletion of *PRDX3* impaired mitochondrial bioenergetics and suppressed mesothelioma growth, while thiostrepton and its clinical formulation RSO-021 produced PRDX3 target engagement and apoptosis in experimental and patient-derived models (Gibson et al., 2026).

Across these studies, PRDX3 shows one of the most consistent cancer-associated phenotypes among the isoforms reviewed. Its predominant localization within the mitochondrial matrix provides a common mechanistic framework, with its effects largely linked to the regulation of mitochondrial rather than cytosolic peroxide homeostasis. Findings in triple-negative breast cancer, gastric cancer, glioblastoma stem-like cells, and mesothelioma are broadly consistent in direction (Chua et al., 2024; Chen et al., 2022; Zhao et al., 2024; Huang et al., 2021), while elevated PRDX3 expression has also been associated with poorer survival in pancreatic ductal adenocarcinoma (Kakehashi et al., 2025). Notably, PRDX3 is currently the only member of the family for which a directly targeted therapeutic agent has progressed to clinical evaluation, distinguishing it from other PRDX isoforms in terms of translational maturity. The disease-control rate in that trial is a preliminary signal, not evidence of efficacy. The study does establish that pro-oxidant targeting of a peroxiredoxin is feasible in patients, which no other family member has yet achieved (Gibson et al., 2026).

#### 3.1.4 PRDX4

PRDX4 is the only PRDX family member that functions in the endoplasmic reticulum (ER) lumen, where its activity is directly coupled to oxidative protein folding and ER stress responses (Fujii et al., 2025). This unique subcellular localization dictates that its role in cancer is highly dependent on tumor stage, ER stress burden and protein secretory demand, rather than simply reflecting an upregulation or downregulation of its expression.

##### 3.1.4.1 Breast cancer

PRDX4 has been implicated in breast cancer progression through both tumor-cell-associated and extracellular mechanisms. Breast cancer cells secrete PRDX4, and PRDX4 depletion reduces their capacity to induce osteoclast formation, while higher PRDX4 expression in primary tumors has been associated with subsequent metastasis (Rafiei et al., 2015). Tiedemann et al. further showed that PRDX4, in cooperation with L-plastin, promotes osteoclastogenesis and bone metastasis by enhancing calcium signaling and nuclear factor of activated T cells 1 (NFATc1) nuclear translocation (Tiedemann et al., 2019). More recently, PRDX4 was reported to promote breast cancer cell proliferation, invasion, and migration and to be associated with neratinib response through a proposed PRDX4–HER2 axis (Jiang et al., 2025). Together, these findings implicate PRDX4 in both breast cancer cell fitness and tumor–bone interactions, although clinical target validation remains lacking.

##### 3.1.4.2 Hepatocellular carcinoma (HCC)

Guo et al. reported that PRDX4 inhibits HCC initiation by reducing oxidative stress, while in established tumors, it promotes cell survival (Guo et al., 2019).

##### 3.1.4.3 Lung cancer

Mizutani et al. found that high PRDX4 expression in lung adenocarcinoma (LUAD) was significantly associated with EGFR mutations, correlating with improved disease-free survival (Mizutani et al., 2019). PRDX4 may serve as a prognostic marker in LUAD, particularly in patients with specific EGFR statuses.

##### 3.1.4.4 Multiple myeloma

Recent evidence has revealed a distinct role of PRDX4 in multiple myeloma. OTUD1 stabilized PRDX4 through deubiquitination, preventing its ER-associated degradation. Increased PRDX4 abundance enhanced immunoglobulin production and promoted accumulation of unfolded proteins, thereby increasing proteasome stress and sensitizing myeloma cells to proteasome inhibitors. These findings identify the OTUD1–PRDX4 axis as a potential therapeutic vulnerability in multiple myeloma (Vdovin et al., 2022).

The PRDX4 findings are best organized by disease stage and endoplasmic reticulum proteostasis rather than by expression alone. In HCC, PRDX4 was reported to restrain tumor initiation while supporting survival of established tumors (Guo et al., 2019); in LUAD, higher expression correlated with EGFR mutation status and improved disease-free survival (Mizutani et al., 2019); and in multiple myeloma, PRDX4 stabilization increased proteotoxic stress and sensitivity to proteasome inhibition (Vdovin et al., 2022). PRDX4 abundance can therefore indicate tissue protection, tumor-cell support, or a treatment-associated vulnerability, and the current evidence does not justify a general PRDX4-inhibition strategy.

#### 3.1.5 PRDX5

PRDX5 plays a crucial role in peroxide detoxification and the maintenance of cellular redox homeostasis. In the cancer models discussed below, elevated expression or activity of PRDX5 has been linked to redox-dependent survival, migration, and resistance to treatment.

##### 3.1.5.1 Non-small-cell lung cancer

Cao et al. discovered that hypomethylation of the *PRDX5* promoter drives NSCLC progression by increasing STAT3 binding and promoting PRDX5 expression, which facilitates cell migration, invasion, and EMT (Cao et al., 2021).

##### 3.1.5.2 Prostate cancer

In castration-resistant prostate cancer (CRPC), the target relevance of PRDX5 has progressed from discovery to preliminary validation. Wang et al. identified PRDX5 as a candidate mediator of CRPC resistance through omics screening (Wang et al., 2024a). Consistently, Tang et al. showed that *PRDX5* depletion increased ROS, induced apoptosis, and enhanced sensitivity to abiraterone and enzalutamide in CRPC cells; partial reversal by N-acetylcysteine supported a ROS-dependent survival mechanism. Pharmacological inhibition of PRDX5 by a stachyose derivative further suppressed CRPC progression, providing additional preclinical support for PRDX5 as a therapeutic target (Wang et al., 2024b). These findings link PRDX5-mediated redox adaptation to androgen receptor signaling inhibitor resistance, but clinical validation is still required (Tang et al., 2025).

##### 3.1.5.3 Colorectal cancer

Ahn et al. showed that PRDX5 promotes EMT in colorectal cancer, enhancing cell proliferation, migration, invasion, and tumor growth (Ahn et al., 2017). *PRDX5* overexpression increased vimentin and Snail/Slug, whereas knockdown reversed EMT-associated changes, supporting a preclinical role rather than a validated biomarker or therapeutic target.

##### 3.1.5.4 Multiple myeloma

Research led by Ren et al. examined the role of PRDX5 in apoptosis induced by epigallocatechin-3-gallate (EGCG) in multiple myeloma cells, where PRDX5 downregulation heightened oxidative stress and cell death (Ren et al., 2011). Overexpressed PRDX5 protected against apoptosis, indicating that PRDX5 is an important mediator of the anticancer effects of EGCG.

PRDX5 is associated with redox-dependent survival, migration, or epithelial–mesenchymal transition in the tumor models reviewed, but the evidence base is small and dominated by cell-based studies. It may be therapeutically relevant in selected settings, though not as a broadly applicable cancer target or biomarker.

#### 3.1.6 PRDX6

PRDX6 has been associated with divergent tumor phenotypes and differs from the other isoforms by combining peroxidase and calcium-independent phospholipase A2 activities. Recent cancer studies particularly implicate it in lipid-peroxide metabolism, ferroptosis resistance, and invasive signaling.

##### 3.1.6.1 Cervical cancer

Hu et al. found that PRDX6 enhances cervical cancer progression by promoting cell proliferation, migration, and invasion while inhibiting apoptosis (Hu et al., 2020).

##### 3.1.6.2 Colorectal cancer

In colorectal cancer models, PRDX6 has been linked to both tumor cell-autonomous and microenvironment-mediated adaptation to oxidative stress. Lagal et al. showed that *PRDX6* knockout increased ROS accumulation and lipid peroxidation, sensitized HCT116 cells to erastin-induced ferroptosis, and reduced their proliferative and invasive phenotypes (Lagal et al., 2024). Independently, *PRDX6* overexpression promoted HCT116 cell proliferation, xenograft growth, and EMT-associated changes, whereas *PRDX6* silencing produced the opposite effects; these phenotypes were accompanied by reciprocal changes in p38 phosphorylation (Chae et al., 2021). Extending these cell-autonomous findings, macrophages and macrophage-derived extracellular vesicles transferred PRDX6 to cancer cells, enhanced glutathione metabolism, reduced lipid peroxidation and mitochondrial ROS, and attenuated ferroptosis-associated mitophagy, thereby increasing ferroptosis resistance (Zheng et al., 2025).

##### 3.1.6.3 Hepatocellular carcinoma

López-Grueso et al. showed that PRDX6 deficiency in HepG2 hepatocarcinoma cells caused mitochondrial dysfunction and cell cycle arrest, reducing tumor proliferation (López-Grueso et al., 2020).

##### 3.1.6.4 Lung cancer

In experimental lung cancer models, PRDX6 increased tumor growth and metastatic phenotypes through its peroxidase and iPLA2 activities, with involvement of MAPK/AP-1 and JAK2/STAT3 signaling (Yun et al., 2014; Yun et al., 2015).

##### 3.1.6.5 Papillary thyroid carcinoma

Nicolussi et al. reported that PRDX1 and PRDX6 were downregulated in papillary thyroid carcinoma, particularly in tumors with BRAF V600E mutations, and that this reduction correlated with aggressive behavior and metastasis (Nicolussi et al., 2014).

PRDX6 is associated with tumor survival, invasion, EMT, and ferroptosis resistance through redox and lipid-peroxide regulation. PRDX6 is elevated or functionally tumor-supportive in cervical, colorectal, hepatocellular, and lung cancer models, whereas it is downregulated in papillary thyroid carcinoma, particularly in BRAF V600E-mutant tumors. This contrast indicates that the relationship between PRDX6 and cancer is tumor-context dependent.

Collectively, the expression patterns, biological effects, and major regulatory mechanisms of PRDX family proteins across different cancer types are summarized in Figure 3 and Table 1.

**FIGURE 3:**
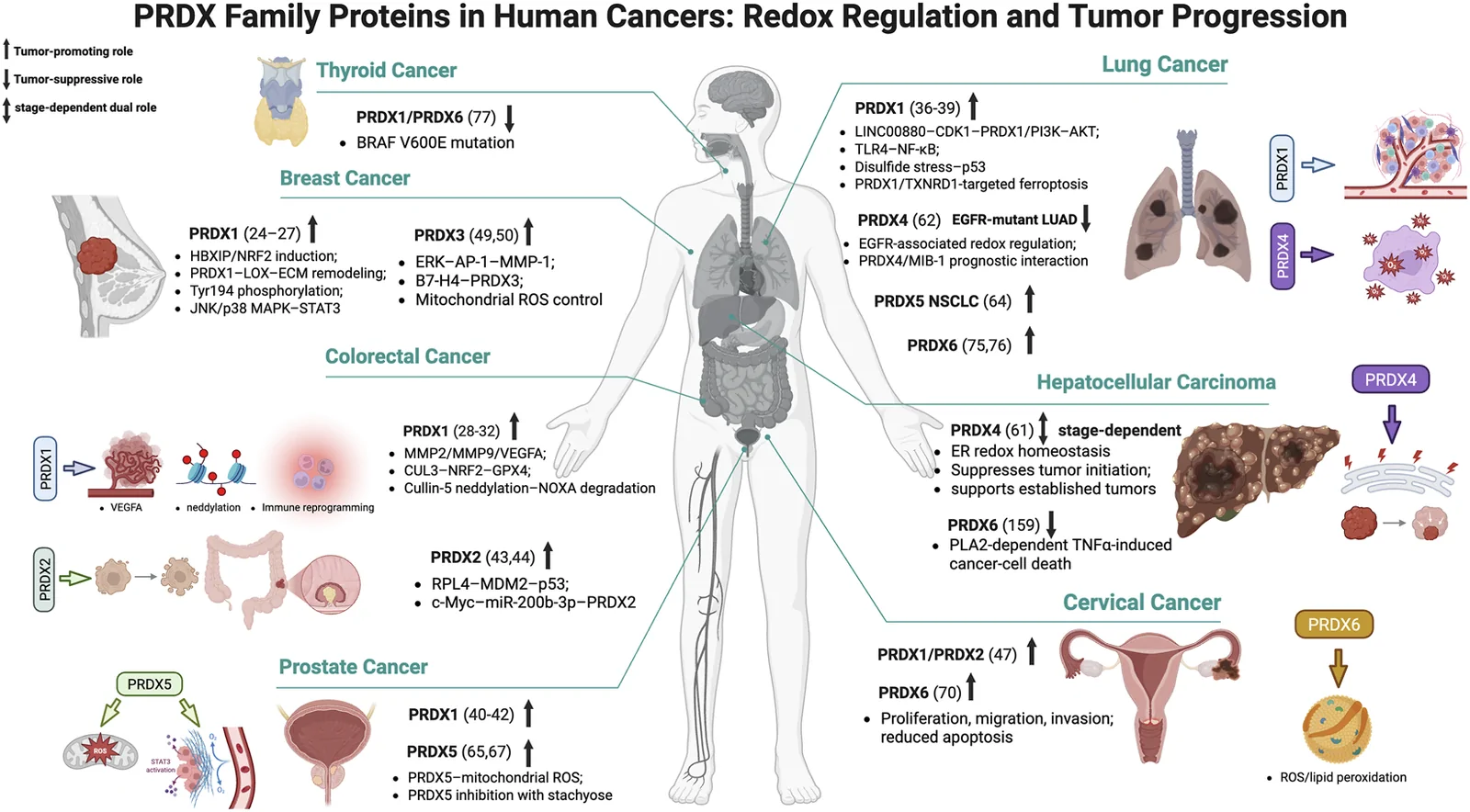
PRDX family proteins in human cancers: redox regulation and tumor progression. Schematic summary of the major tumor-associated roles and molecular mechanisms of selected PRDX isoforms in thyroid, breast, colorectal, lung, hepatocellular, cervical, and prostate cancers. Arrows denote the reported functional roles of PRDXs rather than changes in protein expression: ↑, tumor-promoting role; ↓, tumor-suppressive role; and ↕, context- or stage-dependent dual role. The principal mechanisms shown include redox regulation, extracellular matrix remodeling, epithelial–mesenchymal transition, ferroptosis regulation, immune reprogramming, mitochondrial ROS control, cell survival and apoptosis, and therapy resistance. Numbers in parentheses correspond to references cited in the main text. AR, androgen receptor; ATM, ataxia-telangiectasia mutated; CRPC, castration-resistant prostate cancer; ECM, extracellular matrix; EGFR, epidermal growth factor receptor; EMT, epithelial–mesenchymal transition; ER, endoplasmic reticulum; LUAD, lung adenocarcinoma; NF-κB, nuclear factor-κB; NRF2, nuclear factor erythroid 2-related factor 2; NSCLC, non-small cell lung cancer; PRDX, peroxiredoxin; ROS, reactive oxygen species; STAT3, signal transducer and activator of transcription 3; TLR4, Toll-like receptor 4; TNBC, triple-negative breast cancer.

**TABLE 1:** Expression patterns and functional roles of PRDX family proteins in human cancers.

Cancer type	PRDX protein	Expression/evidence status	Key phenotypes	Principal mechanisms	Evidence/ interpretation	Refs.
Breast cancer	PRDX1	Upregulated (O'Leary et al., 2014)	Survival, anoikis resistance, invasion and metastasis	HBXIP/NRF2–PRDX1; PRDX1–LOX/ECM; Tyr194 phosphorylation; JNK/p38–STAT3	Aggressive phenotype; target hypothesis	(Bajor et al., 2018; Zhou et al., 2022; Attaran et al., 2021; Spínola-Lasso et al., 2023)
Breast cancer (TNBC)	PRDX2	Upregulated (Zhou et al., 2025)	Migration, invasion and EMT	PI3K/AKT/SP1/FN1 axis	Target hypothesis; biomarker unvalidated	(Zhou et al., 2025)
Breast cancer	PRDX3	Upregulated (Chua et al., 2024)	TNBC migration and invasion	ERK–AP-1–MMP-1; B7-H4–PRDX3; mitochondrial ROS control	Metastasis-target hypothesis	(Chua et al., 2024; Chen et al., 2022)
Breast cancer	PRDX4	Upregulated (Jiang et al., 2025)	Osteoclastogenesis and osteolysis; proliferation, invasion, migration, and drug response	Secreted PRDX4/L-plastin-mediated tumor–bone interaction; proposed PRDX4–HER2 axis	Preclinical evidence; clinical target validation lacking	(Rafiei et al., 2015; Tiedemann et al., 2019; Jiang et al., 2025)
Cervical cancer	PRDX1/PRDX2	Upregulated (Lu et al., 2020)	Reduced oxidative stress and apoptosis	ROS buffering; ER-stress attenuation	Chemosensitization hypothesis	(Sun et al., 2024)
Cervical cancer	PRDX6	Upregulated (Hu et al., 2020)	Proliferation, migration and invasion; reduced apoptosis	Mechanism unresolved; associated PCNA/Nanog and BCL-2/BAX changes	Target hypothesis	(Hu et al., 2020)
Colorectal cancer	PRDX1	Upregulated (Song et al., 2024a)	Invasion, angiogenesis, ferroptosis resistance and immune modulation	MMP2/9–VEGFA; Cullin-5–NOXA; CUL3–NRF2–GPX4	Human association; anti-PD-1 sensitization preclinical	(Li et al., 2018; Xu et al., 2021a; Song et al., 2024a; Sun et al., 2025; Xu et al., 2023)
Colorectal cancer	PRDX2	Upregulated (Wang et al., 2021)	Proliferation, invasion and chemoresistance	RPL4–MDM2–p53; c-Myc–miR-200b-3p–PRDX2	Target hypothesis	(Wang et al., 2021; Lv et al., 2017)
Colorectal cancer	PRDX5	Inconsistent expression	EMT, proliferation, invasion and tumor growth	Vimentin/Snail/Slug-associated EMT	Biomarker and target unvalidated	(Ahn et al., 2017)
Colorectal cancer	PRDX6	Inconsistent expression	Ferroptosis resistance, EMT and invasion	Lipid-peroxide detoxification; p38; extracellular-vesicle-mediated mitophagy inhibition	Ferroptosis-target hypothesis	(Lagal et al., 2024; Chae et al., 2021; Zheng et al., 2025)
Endometrial and colorectal cancer stem cells	PRDX3	Upregulated (Song et al., 2017; Song et al., 2015)	Self-renewal, survival, growth and metastasis	FOXM1–PRDX3; mitochondrial redox control	Selected experimental populations only	(Song et al., 2017; Song et al., 2015)
Esophageal cancer	PRDX1	Upregulated (Ren et al., 2013)	Basal tumor fitness; treatment-associated growth suppression	PI3K/AKT; miR-375–PRDX1; FK228-induced PRDX1/p21	Treatment-context dependent	(Wu et al., 2022b; Song et al., 2019; Hoshino et al., 2005)
Gastric cancer	PRDX2	Upregulated (Wang et al., 2020)	ROS-dependent ER/mitochondrial stress and tumor suppression	Celastrol–PRDX2 adduct; ROS accumulation	PRDX2 engagement supported; celastrol nonselective	(Chen et al., 2020)
Gastric cancer	PRDX3	Upregulated (Zhao et al., 2024)	Growth and oxidative-stress resistance	FOXM1–ASF1B–PRDX3 axis	Target hypothesis	(Zhao et al., 2024)
Glioblastoma	PRDX3	No established protein-expression direction	Stem-like cell self-renewal	PHB–PRDX3; mitochondrial ROS suppression	Radiosensitization hypothesis	(Huang et al., 2021)
HCC	PRDX4	Downregulated (Xu et al., 2021b)	Suppresses tumor initiation; supports established tumors	ER redox control	Stage-dependent effects	(Guo et al., 2019)
HCC	PRDX6	Downregulated (Xu et al., 2016)	Mitochondrial dysfunction, G2/M arrest and reduced proliferation	Metabolic and mitochondrial remodeling after PRDX6 loss	HepG2 loss-of-function evidence only	(López-Grueso et al., 2020)
Lung cancer	PRDX1	Upregulated (Kim et al., 2008)	Malignant signaling; radio- and ATM-inhibitor resistance	LINC00880–CDK1–PRDX1/PI3K– AKT; TLR4–NF-κB; disulfide stress–p53; PRDX1/TXNRD1-targeted ferroptosis	Treatment-response determinant	(Feng et al., 2023; Hao et al., 2023a; Li et al., 2025a; Wang et al., 2025a; Kim et al., 2008)
LUAD	PRDX4	Upregulated (Hao et al., 2023b)	EGFR-context-dependent proliferation; longer disease-free survival	EGFR-dependent modulation of PRDX4-mediated growth suppression	Retrospective association; validation required	(Mizutani et al., 2019; Hao et al., 2023b)
NSCLC	PRDX5	Upregulated (Cao et al., 2021)	Migration, invasion and EMT	Promoter hypomethylation–STAT3–PRDX5/ NRF2	Target hypothesis; biomarker unvalidated	(Cao et al., 2021)
Lung cancer	PRDX6	Upregulated (Park et al., 2017)	Tumor growth and invasive phenotypes	Peroxidase/iPLA2–MAPK/AP-1; JAK2/STAT3	Cell and animal models only	(Yun et al., 2014; Yun et al., 2015)
Melanoma	PRDX2	Downregulated (Lee et al., 2013)	Restrains proliferation, migration and metastasis	Src/ERK suppression; E-cadherin/β-catenin stabilization	Tumor-suppressive counterexample	(Lee et al., 2013)
Multiple myeloma	PRDX4	Upregulated (Chen et al., 2025)	Immunoglobulin production and proteotoxic stress	OTUD1-mediated deubiquitination	Proteasome-inhibitor sensitivity determinant	(Vdovin et al., 2022)
Multiple myeloma	PRDX5	Upregulated (Chen et al., 2025)	Resistance to EGCG-induced oxidative death	PRDX5-dependent redox protection	EGCG-sensitivity determinant	(Ren et al., 2011)
PDAC	PRDX3	Upregulated (Kakehashi et al., 2025)	EMT, metastasis and progression	SALL4–FOXM1–PRDX3; mitochondrial ROS suppression	Poor-survival association; validation required	(Kakehashi et al., 2025; Huynh et al., 2018)
Papillary thyroid carcinoma	PRDX1/PRDX6	Downregulated (Nicolussi et al., 2014)	Aggressive behavior and metastasis	BRAF V600E-mediated repression of PRDX1/PRDX6	Prognostic association; performance unvalidated	(Nicolussi et al., 2014)
Pleural mesothelioma	PRDX3	Upregulated (Nelson et al., 2021)	Bioenergetic impairment and apoptosis after targeting	Mitochondrial peroxide detoxification; covalent engagement	Phase I: tolerability/target engagement; efficacy unproven	(Gibson et al., 2026)
Prostate cancer	PRDX1	Upregulated (Riddell et al., 2011)	AR signaling, survival, migration and vascularization	AR transactivation; TLR4/VEGFA; TPD52-enhanced peroxidase activity	Target hypothesis	(Park et al., 2007; Riddell et al., 2011; Dasari et al., 2019)
Prostate cancer	PRDX5	Upregulated (Tang et al., 2025)	CRPC survival and AR-signaling-inhibitor resistance	ROS-dependent survival; sensitization to abiraterone/enzalutamide	Combination-target hypothesis	(Wang et al., 2024a; Tang et al., 2025)

Expression/evidence status indicates the reported direction of PRDX, abundance in human tumor tissue and is cited separately from the functional studies in the Refs. column. Upregulated and Downregulated indicate a consistent direction, Inconsistent expression indicates conflicting reports, and No established protein-expression direction indicates insufficient abundance data. Key phenotypes and Principal mechanisms summarize functional evidence, while Evidence/interpretation defines the strongest supported claim. Unless otherwise specified, entries are preclinical and do not imply validated therapeutic targets or biomarkers. AP-1, activator protein 1; AR, androgen receptor; ATM, ataxia-telangiectasia mutated; CDK1, cyclin-dependent kinase 1; CRPC, castration-resistant prostate cancer; CUL3, cullin 3; ECM, extracellular matrix; EGCG, epigallocatechin-3-gallate; EGFR, epidermal growth factor receptor; EMT, epithelial–mesenchymal transition; ER, endoplasmic reticulum; FOXM1, forkhead box M1; GPX4, glutathione peroxidase 4; iPLA2, calcium-independent phospholipase A2; JAK2, Janus kinase 2; LOX, lysyl oxidase; MMP, matrix metalloproteinase; NF-κB, nuclear factor-κB; NFATc1, nuclear factor of activated T cells 1; NRF2, nuclear factor erythroid 2-related factor 2; PD-1, programmed cell death protein 1; PDAC, pancreatic ductal adenocarcinoma; PHB, prohibitin; PI3K, phosphoinositide 3-kinase; PRDX, peroxiredoxin; ROS, reactive oxygen species; STAT3, signal transducer and activator of transcription 3; TLR4, Toll-like receptor 4; TNBC, triple-negative breast cancer; TPD52, tumor protein D52; TXNRD1, thioredoxin reductase 1; VEGFA, vascular endothelial growth factor A.

#### 3.1.7 Targeting PRDXs for cancer therapy

Targeting PRDXs has emerged as a potential anticancer strategy, primarily by disrupting redox adaptation and increasing oxidative stress in tumor cells. Preclinical studies have identified several compounds targeting PRDX1 and PRDX2, with evidence of increased ROS accumulation, tumor-cell death, and treatment sensitization (Spínola-Lasso et al., 2023; Xu et al., 2023).

Among the PRDX family, PRDX3 has achieved the greatest translational progress. The PRDX3-targeting agent RSO-021 has entered phase I clinical evaluation in pleural mesothelioma, showing tolerability and target engagement, with preliminary disease-control signals but no efficacy data. PRDX3 therefore currently represents the only PRDX isoform to have been evaluated clinically as a therapeutic target (Gibson et al., 2026).

The pharmacological strategies, limitations, and future development of PRDX-targeted therapies are discussed in detail in subsequent sections.

### 3.2 PRDXs in neurodegenerative diseases

PRDXs have been implicated in the pathophysiology of several neurodegenerative diseases, including Alzheimer’s disease (AD), Parkinson’s disease (PD), and amyotrophic lateral sclerosis (ALS) (Szeliga, 2020). In AD, PRDX2 is significantly elevated in the frontal cortex of patients relative to age-matched controls, whereas PRDX1 is unchanged (Krapfenbauer et al., 2003). In experimental systems, PRDX2 helps to mitigate oxidative damage caused by amyloid-β accumulation by reducing ROS levels and protects neurons from oxidative stress-induced apoptosis (Jin et al., 2022). Additionally, PRDX6 has been shown to protect neuronal cells from lipid peroxidation, a process closely associated with the neurodegenerative changes observed in AD (Power et al., 2008).

PRDX2 appears to play a predominantly neuroprotective role in PD by preserving intracellular redox homeostasis. However, its antioxidant function is compromised by nitrosative stress. S-nitrosylated PRDX2 accumulates in PD brains and exhibits impaired peroxidase activity, a change that has been linked to oxidative neuronal injury (Fang et al., 2007). This modification can be reversed by sulfiredoxin in human induced pluripotent stem cell (iPSC)-derived dopaminergic neurons, restoring PRDX2 function and attenuating nitrosative stress (Sunico et al., 2016). Consistent with these findings, experimental overexpression of *PRDX2* suppresses ROS accumulation and apoptosis in 1-methyl-4-phenylpyridinium (MPP^+^)-treated SH-SY5Y cells, supporting a protective role against dopaminergic neurodegeneration in this model (Liu et al., 2023). Mitochondrial dysfunction and oxidative stress are central features of PD (Hattori and Sato, 2024). PRDX3 is a mitochondria-resident peroxidase that contributes to mitochondrial redox homeostasis by removing hydrogen peroxide and limiting oxidative damage to mitochondrial DNA (Liu et al., 2016). In a toxin-induced animal model of PD, brain-targeted *PRDX3* gene delivery protected dopaminergic neurons, reduced neurodegeneration, and improved motor performance (Villa-Cedillo et al., 2025). Recent data in PD further support a ferroptosis-centered role for PRDX6. In models of PLA2G6-associated parkinsonism, loss of PLA2G6 destabilized PRDX6 and disrupted the PRDX6/FTH1/GPX4 axis, leading to iron accumulation, enhanced lipid peroxidation, and dopaminergic neuronal ferroptosis. Restoration of PRDX6 alleviated ferroptotic vulnerability, suggesting that PRDX6-dependent control of iron handling and lipid peroxide defense may be relevant to neurodegeneration (Li T. et al., 2025). However, these genetically defined or toxin-induced models impose the redox lesion experimentally; whether the same sequence operates in idiopathic PD remains uncertain.

ALS is characterized by degeneration of motor neurons, in which oxidative stress contributes to disease progression. PRDX2 immunoreactivity is altered in surviving motor neurons of patients with sporadic and superoxide dismutase 1 (SOD1)-mutated familial ALS and in SOD1-mutant animal models (Kato et al., 2005) and PRDX1 is among the antioxidant proteins upregulated in motor neuron-like cells expressing mutant SOD1 (Allen et al., 2003); a direct interaction between PRDX1 and mutant SOD1 has not, however, been demonstrated. The direction of effect appears to depend on the cell type involved: peroxiredoxins secreted by Schwann cells protect motor neurons against hydrogen peroxide (Yamamuro-Tanabe et al., 2023a), whereas PRDX6 upregulation in spinal astrocytes drives their conversion to a neurotoxic A1 phenotype (Yamamuro-Tanabe et al., 2023b).

These studies support an association between PRDX-dependent redox regulation and neurodegenerative pathology, with context-specific functional effects in cellular and animal models. They do not, however, establish the extent to which PRDX dysregulation is a primary cause of human neurodegeneration. Altered PRDX expression or oxidation may represent a disease-promoting mechanism, a compensatory response to oxidative stress, or a downstream consequence of neuronal injury. The therapeutic record points the same way: non-selective antioxidant supplementation has produced little consistent clinical benefit in AD or PD (Chang and Chen, 2020; Li et al., 2026), suggesting that oxidative stress is neither a simple nor a readily modifiable cause of these diseases. Distinguishing among the possibilities above will therefore require longitudinal human studies and disease-relevant models with isoform-specific genetic or pharmacological manipulation. Until such data exist, PRDX modulation remains a hypothesis to be tested. The limited benefit of bulk antioxidant supplementation is itself an argument for the isoform- and compartment-resolved strategies considered below.

### 3.3 PRDXs in cardiovascular diseases

PRDXs have context-dependent relationships with cardiovascular diseases, including atherosclerosis, myocardial infarction, and ischemia-reperfusion injury, rather than uniformly protective effects (Li et al., 2020). In apolipoprotein E (ApoE)-deficient mouse models, loss of *Prdx1* or *Prdx2* increased endothelial activation, leukocyte recruitment, and atherosclerotic plaque burden, while macrophage PRDX1 also supported lipophagic cholesterol efflux and PRDX2 constrained inflammatory phenotypic changes in vascular smooth muscle cells (Kisucka et al., 2008; Park et al., 2011; Jeong et al., 2018; Li et al., 2021). These experiments support anti-atherogenic functions for intracellular PRDX1/PRDX2 in vascular and myeloid compartments, but they do not establish that PRDX2 directly prevents low-density lipoprotein oxidation. Localization also matters: extracellular PRDX5 promoted endothelial TLR4/MyD88 signaling and plaque inflammation in a recent ApoE-deficient model (Kweon et al., 2025). Thus, the net vascular phenotype depends on isoform, cellular source, and extracellular release.

In the myocardium, the strongest mechanistic evidence concerns control of mitochondrial peroxide homeostasis and quality control. *Prdx3*-deficient mice developed age-associated cardiac hypertrophy and dysfunction with impaired PINK1-dependent mitophagy, whereas matairesinol attenuated pressure overload-induced cardiac remodeling in a *PRDX1*-dependent manner, and *Prdx5* overexpression mitigated doxorubicin-induced cardiac hypertrophy and fibrosis in separate mouse models (Sonn et al., 2022; Zhang et al., 2024; Hu et al., 2025). Several PRDX isoforms can therefore modify cardiac stress responses. However, the evidence is derived mainly from genetic or viral-vector manipulations in animals. It does not establish a causal role in human heart failure.

During ischemia/reperfusion, *Prdx3* loss increased mitochondrial H_2_O_2_, mitochondrial fragmentation, defective mitophagy, lysosomal dysfunction, and myocardial injury in cardiomyocyte and mouse models, while PRDX1 attenuated hypoxia/reoxygenation-associated MAPK activation and apoptosis in rat-derived systems (Park et al., 2025; Guo et al., 2018). Both studies support a cardioprotective capacity under defined experimental conditions; however, one relied heavily on H9c2 cells, and neither demonstrates that increasing PRDX activity is safe or effective in patients. The evidence therefore links PRDX-dependent redox regulation and organelle quality control to injury severity without establishing a clinically actionable target.

Recent work extends this picture to metabolic heart failure and circulating biomarkers. In a mouse model of heart failure with preserved ejection fraction, PINK1-dependent maintenance of PRDX2 reduced lipotoxic apoptosis and improved diastolic function (Zha et al., 2025). In humans, by contrast, higher circulating PRDX4 was associated with incident heart failure in a large community cohort and was elevated in a small acute coronary syndrome cohort (Suthahar et al., 2025; Doughty et al., 2024). Because these studies evaluated associations and risk stratification rather than PRDX4 activity within the myocardium, elevated PRDX4 is better interpreted as a marker of systemic oxidative stress than as evidence of cardioprotection. Overall, the cardiovascular literature is mechanistically suggestive but still predominantly preclinical, with observational human data that do not establish causality or therapeutic efficacy.

### 3.4 PRDXs in inflammatory and immune disorders

PRDXs influence inflammatory disease mechanisms through isoform- and compartment-dependent pathways. Intracellular PRDXs regulate peroxide-dependent signaling, whereas extracellular PRDXs released from damaged cells can function as inflammatory mediators by activating Toll-like receptors or complement pathways (Riddell et al., 2010; Poncin et al., 2021; Park et al., 2021). Intracellular PRDX1 can conversely suppress TLR4-dependent NF-κB activation by inhibiting TRAF6 ubiquitin ligase activity (Min et al., 2018), illustrating that PRDXs may exert opposing effects depending on their localization and molecular interactions.

In rheumatoid arthritis (RA), PRDX2 is enriched in lymphocytes, particularly IL-17-secreting cells, while PRDX4 is increased in hyperplastic synovial tissue (Szabó-Taylor et al., 2012; Aihaiti et al., 2022). *PRDX4* silencing suppresses the proliferative and invasive phenotype of RA fibroblast-like synoviocytes through PI3K/AKT signaling (Aihaiti et al., 2022). In experimental arthritis, *Prdx6* overexpression aggravates inflammation through JNK-associated activation of NF-κB and AP-1 (Kim et al., 2015), whereas PRDX2 inhibition by capsaicin induces ROS-dependent synoviocyte apoptosis and attenuates disease (He et al., 2025). Selected PRDX isoforms therefore appear to sustain pathogenic immune-cell and synoviocyte phenotypes beyond any antioxidant protection they provide.

In systemic lupus erythematosus, altered PRDX levels have mainly been reported as markers of disrupted redox homeostasis, without evidence that they directly regulate autoantibody production or immune-complex injury (Krata et al., 2021; Kim et al., 2024). PRDX5 also shows context-dependent effects: it suppresses LPS-induced IL-6 production through JAK2–STAT5 interactions (Choi et al., 2013) but promotes M1 macrophage polarization through TLR4/NF-κB signaling in experimental autoimmune prostatitis (Wu et al., 2025). PRDXs are therefore best regarded as context-dependent regulators of inflammatory signaling rather than uniformly protective antioxidants or established anti-inflammatory targets.

### 3.5 PRDXs in infectious diseases

PRDXs have bidirectional roles in infection because host enzymes can protect tissues while also limiting microbicidal oxidants. In THP-1 macrophage models, loss of *PRDX3* or *PRDX6* increased mitochondrial ROS and enhanced killing of *Salmonella Typhimurium*; PRDX6 also interrupted the TRAF6-ECSIT complex and reduced TLR4-dependent NF-κB activation (Lee et al., 2016; Min et al., 2017). In contrast, *Prdx1*-deficient mice were more susceptible to *Mycobacterium tuberculosis*, with impaired IL-12/IFN-γ and nitric oxide responses (Matsumura et al., 2016). These divergent results show that host PRDXs cannot be classified as uniformly beneficial or detrimental during infection; the outcome depends on isoform, pathogen, and the balance between antimicrobial ROS and immunopathology.

Pathogens may also modify host PRDXs, although biochemical interaction does not necessarily determine disease outcome. The Pla protease of *Yersinia pestis* cleaved and inactivated host PRDX6 *in vitro* and reduced extracellular PRDX6 in infected lungs, yet *Prdx6* deficiency did not alter bacterial burden, inflammation, lung injury, or survival in that model (Zimbler et al., 2016). This negative result is important: it cautions against inferring virulence relevance from substrate cleavage alone.

PRDXs are also involved in microbial antioxidant defenses. Many pathogenic bacteria, such as *H. pylori*, express PRDX homologs that help them survive the oxidative environment of the host. *H. pylori* PRDX, for example, protects the bacterium from the oxidative burst produced by host immune cells, allowing it to establish chronic infection in the gastric mucosa (Wang et al., 2006a; Wang et al., 2006b). Microbial PRDXs are therefore potential antimicrobial targets because their inhibition could make pathogens more susceptible to oxidative killing by the host immune system.

The dual role of PRDXs in host defense and in microbial survival complicates their interpretation in infectious diseases (Kusakisako et al., 2018; Mastronicola et al., 2014). Because their catalytic architecture is conserved, active-site-directed inhibitors may cross-react with human PRDXs. Selectivity will therefore probably depend on structural features outside the catalytic center, which remain incompletely characterized. The preferred intervention may also vary among infections: enhancing host PRDX activity could limit immunopathology, whereas inhibiting a host isoform exploited by a pathogen, or directly inhibiting microbial PRDXs, could facilitate pathogen clearance. No criteria for choosing between these strategies have been proposed.

## 4 Therapeutic targeting of peroxiredoxins

### 4.1 Potential as therapeutic targets

PRDXs have emerged as therapeutic targets owing to their central role in maintaining cellular redox homeostasis and modulating various signaling pathways associated with disease processes (Hampton et al., 2018). Targeting PRDXs presents a feasible approach for treating a range of diseases, including cancer, neurodegenerative diseases, cardiovascular diseases, and inflammatory disorders (Szeliga, 2020; Robinson et al., 2010; Jeong et al., 2021). The overexpression of certain PRDX isoforms in various diseases, such as PRDX1 in several cancer types and PRDX2 in neurodegenerative diseases, indicates their involvement in disease pathogenesis and supports their potential as therapeutic targets (Szeliga, 2020; Guan et al., 2024).

The feasibility of targeting PRDXs for disease treatment lies in their role as regulators of oxidative stress (Winterbourn, 2025; Bertoldi, 2016). By modulating PRDX activity, it may be possible to restore redox balance and suppress pathological processes that are driven by oxidative damage (Wang et al., 2014). For example, inhibiting overexpressed PRDXs in cancer cells could enhance the efficacy of chemotherapeutic agents by increasing intracellular ROS levels, thereby inducing cancer cell death (Bajor et al., 2018; Sun et al., 2024). Conversely, enhancing PRDX activity in neurodegenerative diseases could protect neuronal cells from oxidative stress-induced damage, thereby slowing disease progression (Villa-Cedillo et al., 2025).

However, there are significant challenges associated with targeting PRDXs. One major challenge is the ubiquitous expression of PRDXs and their essential role in normal cellular function. Modulating PRDX activity therefore raises the possibility of on-target disruption of redox balance in healthy tissues and unintended toxicity; accordingly, isoform selectivity, target engagement, and safety require direct evaluation, and clinical evidence remains limited to early-phase testing (Gibson et al., 2026; Forshaw et al., 2019). Moreover, the structural similarity among PRDX isoforms complicates the development of isoform-specific inhibitors or activators, while partial functional overlap may limit the therapeutic efficacy of targeting a single PRDX isoform. Therefore, while targeting PRDXs holds significant promise, careful consideration of the benefits and challenges is essential for the successful development of PRDX-targeted therapies.

### 4.2 Small molecule inhibitors and activators

The development of small molecule inhibitors and activators targeting PRDXs is a rapidly evolving field, with multiple promising compounds under investigation in preclinical settings. Small molecule inhibitors of PRDXs are primarily being explored for their potential in cancer therapy, aiming to enhance intracellular oxidative stress in cancer cells, thereby inducing apoptosis (Park et al., 2016). By contrast, small molecule activators of PRDXs are being investigated for their potential neuroprotective effects, particularly in the context of neurodegenerative diseases where enhanced antioxidant defense is beneficial.

Conoidin A was identified as a covalent inhibitor of PRDX2 (Haraldsen et al., 2009) and subsequently reduced the growth of glioblastoma cell lines and patient-derived cells through ROS accumulation (Szeliga et al., 2023). Its *in vivo* efficacy and family-wide selectivity remain undefined, and no study has shown that it enhances conventional chemotherapy. Adenanthin was initially reported to inhibit PRDX1 and PRDX2 and induce differentiation of leukemic cells (Liu et al., 2012). Subsequent studies, however, demonstrated broader thiol reactivity and substantially greater inhibition of thioredoxin reductase, indicating that adenanthin should not be regarded as a selective PRDX inhibitor and that its cellular effects cannot be attributed specifically to PRDX inhibition (Soethoudt et al., 2014).

More recent studies provide more isoform-resolved evidence. A structure-guided celastrol urea derivative covalently bound PRDX1 Cys173, showed weak inhibition of PRDX2-PRDX6, induced ROS-linked apoptosis in colorectal cancer cells, and suppressed xenograft growth (Li et al., 2024). Okanin directly bound PRDX5, inhibited its enzymatic activity, promoted its degradation, and induced apoptosis and ferroptosis in colorectal cancer models (Zhao et al., 2025). A caproate derivative of the prebiotic stachyose provided a second PRDX5-directed approach by inhibiting PRDX5 activity, disrupting the PRDX5–NRF2 interaction, and lowering NAD(P)H quinone dehydrogenase 1 (NQO1), leading to quinone radical accumulation and apoptosis of drug-tolerant persister cells in castration-resistant prostate cancer (Wang et al., 2024b). Thiostrepton-derived fragments covalently cross-linked the catalytic cysteines of PRDX3 while eliminating several alternative targets of the parent compound (Nelson et al., 2025). Most notably, locally administered intrapleural RSO-021, a thiostrepton formulation, showed acceptable tolerability and preliminary disease-control signals in a small phase I trial in relapsed pleural mesothelioma (Gibson et al., 2026). Selected PRDX isoforms are therefore druggable, but only the PRDX3 program has reached early clinical testing, and efficacy there is unproven.

Pharmacological activation of PRDXs may offer a complementary strategy in neurological injury. Obovatol enhanced PRDX2 antioxidant activity and attenuated microglia-mediated neuroinflammation (Ock et al., 2010). Recently, structure-guided optimization of salvianolic-acid scaffolds yielded LC-PDA-01, a direct PRDX1 activator that provides a tractable lead for further pharmacological development (Zhu et al., 2026). PRDX activation is therefore a plausible neuroprotective strategy, although it has not been tested in chronic neurodegenerative disease.

Despite the progress in developing PRDX-targeting drugs, there are challenges that need to be addressed. One major challenge is the specificity of these compounds, as many small molecules that target PRDXs also affect other antioxidant enzymes, leading to off-target effects (Soethoudt et al., 2014). Additionally, the development of drug resistance is a concern, particularly in cancer therapy, where tumor cells may upregulate compensatory antioxidant pathways to counteract the effects of PRDX inhibitors (Gibson et al., 2026). Future research should focus on improving the specificity of PRDX-targeting compounds and exploring combination therapies to overcome resistance mechanisms. A summary of the agents, mechanisms of action, disease contexts, and current evidence levels of PRDX-targeted therapeutic strategies is presented in Table 2.

**TABLE 2:** Therapeutic targeting of peroxiredoxins: agents, mechanisms, and current evidence.

Agent/approach	Target isoform	Mode of action	Disease context	Highest evidence	Evidence/interpretation	Refs.
Small molecule inhibitors						
Celastrol	PRDX1	Covalent engagement; ROS-linked apoptosis	Colorectal cancer	*In vitro*	Multi-target quinone methide; not PRDX1-selective	(Xu et al., 2023)
Celastrol urea derivative	PRDX1	Covalent binding to Cys173; ROS-linked apoptosis	Colorectal cancer	*In vitro*; xenograft	Only weak activity against PRDX2-PRDX6, indicating isoform selectivity; preclinical	(Li et al., 2024)
CM728	PRDX1	PRDX1 inactivation; ROS accumulation; MAPK activation; STAT3 inhibition	Triple-negative breast cancer	*In vitro*	Chemical-proteomic target; family-wide selectivity untested	(Spínola-Lasso et al., 2023)
Glaucocalyxin A	PRDX1/TXNRD1	Dual engagement; autophagy-mediated ferroptosis	Non-small-cell lung cancer	*In vitro*	Effects cannot be assigned to PRDX1 alone	(Wang et al., 2025a)
Conoidin A	PRDX2	Covalent inhibition; ROS accumulation	Glioblastoma	*In vitro*	Family selectivity and *in vivo* efficacy unresolved	(Haraldsen et al., 2009; Szeliga et al., 2023)
Celastrol	PRDX2	Covalent engagement; ROS-linked ER/mitochondrial stress	Gastric cancer	*In vitro*	Multi-target compound; not PRDX2-selective	(Chen et al., 2020)
Capsaicin	PRDX2	PRDX2 inhibition; ROS-dependent synoviocyte apoptosis	Rheumatoid arthritis	*In vitro*; animal model	PRDX2 selectivity unestablished	(He et al., 2025)
Adenanthin	PRDX1/PRDX2	Thiol reactivity; leukemic-cell differentiation	Leukemia	*In vitro*	More potent against thioredoxin reductase; not a selective PRDX inhibitor	(Liu et al., 2012; Soethoudt et al., 2014)
Thiostrepton	PRDX3	Catalytic-cysteine cross-linking	Mesothelioma	*In vitro*; patient-derived explants	Parent compound engages additional targets	(Gibson et al., 2026)
Thiostrepton-derived fragment	PRDX3	Catalytic-cysteine cross-linking	Cancer models	Biochemical; *in vitro*	Improved selectivity; preclinical only	(Nelson et al., 2025)
RSO-021	PRDX3	Catalytic-cysteine cross-linking; thiostrepton formulation	Relapsed pleural mesothelioma	Phase I clinical trial	Tolerability and target engagement reported; efficacy unproven	(Gibson et al., 2026)
Okanin	PRDX5	Direct inhibition/degradation; apoptosis and ferroptosis	Colorectal cancer	*In vitro*; animal model	Family-wide selectivity unreported	(Zhao et al., 2025)
Stachyose caproate	PRDX5	PRDX5 inhibition; disruption of PRDX5–NRF2 interaction; NQO1 downregulation	Castration-resistant prostate cancer	*In vitro*; animal model	Preclinical pharmacokinetic/tolerability data; family selectivity unestablished	(Wang et al., 2024b)
Small molecule activators						
Obovatol	PRDX2	Enhances antioxidant activity; limits microglial inflammation	Neuroinflammation	*In vitro*; animal model	Directness and isoform selectivity incompletely defined	(Ock et al., 2010)
LC-PDA-01	PRDX1	Direct activator derived from salvianolic-acid scaffolds	Neurological injury	*In vitro*	Efficacy in chronic neurodegenerative disease unproven	(Zhu et al., 2026)
Genetic approaches						
Brain-targeted PRDX3 delivery	PRDX3	Gene overexpression	Toxin-induced Parkinson’s disease model	Animal model	Proof of principle; no replication or clinical evidence	(Villa-Cedillo et al., 2025)
Combination strategies						
Co-targeting compensatory redox pathways	PRDX1/PRDX3	Limits Ref-1- or SLC7A11-associated redox adaptation	Pancreatic cancer; mesothelioma	Preclinical mechanistic evidence	Combination efficacy, selectivity, and safety remain unestablished	(Gibson et al., 2026; Kiran et al., 2025)

Evidence level denotes the most advanced experimental or clinical system reported and does not imply effect magnitude or therapeutic efficacy. Except for RSO-021, all listed strategies remain preclinical; RSO-021, has undergone phase I clinical evaluation, but clinical efficacy has not been established. Isoform selectivity is considered unestablished unless selectivity across the PRDX, family was directly evaluated. The combination-strategy entry represents a mechanistic therapeutic concept rather than a clinically validated intervention. ER, endoplasmic reticulum; MAPK, mitogen-activated protein kinase; NQO1, NAD(P)H quinone dehydrogenase 1; NRF2, nuclear factor erythroid 2-related factor 2; PRDX, peroxiredoxin; Ref-1, redox factor 1; ROS, reactive oxygen species; SLC7A11, solute carrier family 7 member 11; STAT3, signal transducer and activator of transcription 3; TXNRD1, thioredoxin reductase 1.

### 4.3 Gene therapy approaches

Genetic modulation of PRDX expression has so far been pursued experimentally rather than clinically. Gene-editing technologies such as clustered regularly interspaced short palindromic repeats (CRISPR)/CRISPR-associated protein 9 (Cas9) could in principle enable isoform-selective up- or downregulation beyond that currently achieved with small molecules (Hussain et al., 2019). By specifically targeting the genes encoding PRDX isoforms, it is possible to either upregulate or downregulate their expression, depending on the therapeutic need. In existing studies, genetic depletion has primarily served to establish PRDX dependency rather than to represent a therapeutic modality itself.

The most direct evidence comes from Parkinson’s disease, where brain-targeted delivery of PRDX3 protected dopaminergic neurons, reduced neurodegeneration, and improved motor performance in a toxin-induced model (Villa-Cedillo et al., 2025). Although this study provides proof of principle, the evidence is currently limited to a single animal model. Comparable gene-delivery studies have not been reported for other PRDX isoforms, other neurodegenerative conditions, or cardiovascular disease, where the proposed benefits of PRDX overexpression are based primarily on transgenic and pharmacological evidence rather than on the delivery of a therapeutic gene construct.

Two practical obstacles are commonly cited. The first is delivery of gene-editing tools to specific tissues or cell types, as systemic delivery could lead to off-target effects and unintended consequences (Song Z. et al., 2024; Naghib et al., 2024). Second, the long-term consequences of altering PRDX expression are unknown (Lee et al., 2024). Before PRDX-directed gene therapy can be considered translationally realistic, studies must establish isoform-specific causality, reproducible benefit in disease-relevant models, and clinically relevant safety and target engagement. Accordingly, gene-based modulation of PRDXs should be presented as a future research direction, not as a currently actionable treatment.

## 5 Challenges and opportunities

### 5.1 Challenges in therapeutic targeting

Therapeutic targeting of PRDXs faces several challenges, as outlined in Section 4.1. PRDXs are widely expressed and contribute to normal redox homeostasis. Their structural similarity complicates isoform-selective targeting (Kim and Jang, 2019), and inhibition of one isoform may be buffered by compensatory antioxidant mechanisms (Melhem et al., 2017). Their context-dependent functions add a further layer of complexity, as PRDXs can support either cell survival or cell death depending on the cellular and redox environment (Song et al., 2017; Guo et al., 2019). Although PRDXs frequently promote tumor survival by limiting oxidative stress, this pattern is not universal: PRDX2 suppresses melanoma metastasis (Lee et al., 2013), high PRDX4 expression is associated with better disease-free survival in lung adenocarcinoma (Mizutani et al., 2019), and PRDX5 deficiency in macrophages promotes lung cancer growth (Seong et al., 2021). The consequences of targeting a PRDX therefore cannot be inferred from its expression level alone, but must be evaluated in the context of the specific isoform, cell type, and disease stage.

### 5.2 Opportunities for novel drug development and combined therapeutic strategies

Recent work indicates that chemical selectivity can be improved, as activity-based protein profiling has identified compounds with isoform-selective engagement (Li et al., 2024; Nelson et al., 2025). Notably, a minimal thiostrepton fragment retained PRDX3 inhibition while eliminating three off-target interactions of the parent compound, suggesting that PRDX selectivity can be improved through rational chemical optimization rather than presumed from initial activity profiles (Nelson et al., 2025).

Delivery strategies provide another means of improving therapeutic specificity. As discussed in Section 3.1.3, local intrapleural administration showed acceptable tolerability in the mesothelioma trial, supporting localized exposure as a practical approach when tumor anatomy permits (Gibson et al., 2026). For less accessible tumors, nanoparticle-based delivery or antibody–drug conjugates could extend this concept by increasing tumor-selective exposure while limiting systemic effects (Naghib et al., 2024).

Combination strategies may also be important. Inhibition of a single PRDX isoform can be buffered by parallel antioxidant pathways, providing a rationale for simultaneously targeting the glutathione or thioredoxin systems or for combining PRDX inhibition with ROS-inducing chemotherapy (Gibson et al., 2026; Kiran et al., 2025). The identification of SLC7A11 as a mediator of thiostrepton resistance offers a specific example of such adaptive redox compensation and a potential basis for rational combination therapy (Gibson et al., 2026).

### 5.3 The potential of PRDXs as biomarkers for prognosis and treatment response

Beyond their role as therapeutic targets, PRDXs hold considerable promise as biomarkers for disease prognosis and treatment response. The expression levels of certain PRDX isoforms are often altered in various diseases, and these changes may provide valuable information about disease progression, patient prognosis, and response to therapy. The direction of association is not uniform, however, and differs between tumor types. High PRDX1 is associated with poorer survival in colorectal cancer (Fischer et al., 2018), and high PRDX3 with poorer survival in pancreatic ductal adenocarcinoma (Kakehashi et al., 2025), whereas higher PRDX4 expression has been associated with more favorable disease-free survival in lung adenocarcinoma (Mizutani et al., 2019; Shioya et al., 2018), and reduced PRDX1 and PRDX6 with more aggressive papillary thyroid carcinoma (Nicolussi et al., 2014). A biomarker application must therefore be defined for a specific isoform in a specific tumor type rather than for the family as a whole.

The most developed example comes from prostate cancer. Serum PRDX5, measured together with GPX4, ubiquinone oxidoreductase subunit S4 (NDUFS4), and thioredoxin reductase 2 (TXNRD2), predicted the later development of castration resistance in a cohort of 482 patients (Wang R. et al., 2025), and PRDX5 expression in tumor tissue was higher in advanced disease (Tang et al., 2025). This is currently the only PRDX measurement validated in a clinical cohort of this size, and it illustrates the scale of validation required before other isoforms can be used clinically.

In neurodegenerative diseases, PRDXs have been investigated primarily as blood-based biomarkers, although the available evidence remains limited to small cohorts. Serum PRDX3 was reduced in genetic carriers of Parkinson’s disease, including individuals without motor symptoms (Ng et al., 2023). In Alzheimer’s disease, oxidatively modified PRDX2 and PRDX6, rather than total protein levels, were elevated in plasma and erythrocytes, with the extent of modification increasing with clinical dementia rating (Yoshida et al., 2009). The two studies differ in both the direction and the nature of the PRDX change, which is itself an argument against using protein abundance alone as a biomarker readout. However, neither finding has been independently replicated, and no PRDX-based biomarker has yet been validated for clinical use in neurodegenerative disease.

Moreover, PRDXs could be used as biomarkers for assessing the effectiveness of PRDX-targeted therapies. For example, in patients receiving a PRDX inhibitor as part of cancer treatment, measuring changes in PRDX expression or activity could provide an early indication of treatment response. If PRDX levels decrease following treatment, it may suggest that the therapy is effectively targeting the intended pathway and that the patient is likely to benefit from continued treatment.

The development of reliable assays for detecting and quantifying PRDX levels in biological samples is required before PRDXs can be used as biomarkers. Most studies focus on measuring the PRDX protein rather than its activity. The catalytic state, hyperoxidation, and oligomeric form of PRDX can vary independently of its abundance. Currently, standardized assays that can distinguish these states are not available. Until such assays are developed, comparing PRDX measurements across different studies will be challenging, and their clinical application will remain in an exploratory phase.

## 6 Future perspectives

A central priority for future research is to move from descriptive associations toward establishing causality. Although numerous studies have documented altered PRDX expression in disease, far fewer have determined the functional consequences of these changes. The same isoform may exert protective or detrimental effects depending on its subcellular localization, functional activity, and disease stage, none of which can be adequately captured by protein abundance alone. Future studies should therefore focus on distinguishing cause from consequence through isoform-specific genetic models, mutants that separate catalytic from non-catalytic functions, spatially resolved analyses, and longitudinal human studies. Such approaches will be essential for distinguishing primary disease mechanisms from compensatory or secondary responses.

Therapeutic development presents an additional challenge. Because PRDX inhibition can increase ROS levels, and many compounds produce similar effects through unrelated mechanisms, changes in oxidative stress alone do not demonstrate direct PRDX engagement. The first clinical evaluation of a PRDX-directed inhibitor supports the druggability of this protein family, although its local administration in a confined compartment also highlights the challenges of systemic targeting. Further translation will depend on improving isoform selectivity, achieving adequate tissue exposure, and determining whether compensatory antioxidant pathways limit therapeutic responses. In comparison, pharmacological activation of PRDXs remains at an earlier stage of development.

Biomarker development requires a similar shift from abundance toward functional measures. Protein levels alone may provide limited information because catalytic activity, oxidation state, and oligomeric status can vary independently of expression and more directly reflect PRDX function. Current studies have identified several candidate PRDX-related biomarkers, but clinical validation remains limited. Prospective cohorts, standardized assays, independent validation, and longitudinal sampling will therefore be required to determine whether PRDX measurements provide additional prognostic or predictive value beyond established clinical tools.

## 7 Conclusion

The PRDX family is a group of multifunctional redox regulators that maintain cellular homeostasis and influence disease progression. Beyond their classical antioxidant function, PRDXs are now recognized as context-dependent modulators of redox signaling, mitochondrial integrity, inflammatory responses, ferroptosis sensitivity, and tumor adaptation. Their diverse and sometimes opposing roles across cancer, neurodegenerative, cardiovascular, inflammatory, and infectious diseases indicate that PRDX biology must be interpreted at the level of individual isoforms and specific diseases.

Accumulating evidence suggests that PRDXs are being investigated as therapeutic targets and potential biomarkers. However, their clinical exploitation will require a more precise understanding of isoform specialization, subcellular localization, compensatory interactions, and context-dependent protective versus pathogenic functions. Future progress in PRDX research will depend on integrating mechanistic studies with translational strategies, including selective drug development, combination therapies, and biomarker-guided precision medicine. With these advances, PRDXs may become clinically actionable regulators that link oxidative stress to disease-specific outcomes.
